# Randomized Controlled Trial to Compare AmnioExcel Human Amniotic Allograft in Weekly Versus Biweekly Treatment of Diabetic Foot Ulcers

**DOI:** 10.1177/15347346241276697

**Published:** 2025-01-09

**Authors:** Lawrence A. Lavery, Mehmet A. Suludere, Kathryn Raspovic, Peter Andrew Crisologo, Matthew J. Johnson, Arthur N. Tarricone

**Affiliations:** 1 Department of Plastic Surgery, 12334 University of Texas Southwestern Medical Center, Dallas, Texas, USA; 2 Department of Orthopaedic Surgery, 12334 University of Texas Southwestern Medical Center, Dallas, Texas, USA

**Keywords:** ulcer, amniotic membrane, infection, foot, peripheral arterial disease, osteomyelitis

## Abstract

Our objective was to compare clinical outcomes in diabetic foot ulcers (DFU) treated with AmnioExcel^®^ applied weekly (AMX1) or biweekly (AMX2) over a 12-week evaluation period. This randomized clinical trial evaluated 40 people with UT 1A and 1D DFUs >30 days but less than 6 months duration and age >21 years. We excluded patients with untreated osteomyelitis, gangrene, widespread malignancy, or active substance abuse. Patients received amniotic tissue either once a week or every other week. We used a 3D measurement device (inSight, eKare, Fairfax, Virginia)”. There was no difference in the incidence of healing (AMX1 30.0% vs AMX2 50.0%, p = 0.20), time to heal (69.3 ± 30.3 vs 45.8 ± 25.6 days, p = 0.15), or incidence of infection (AMX1 35.0% vs AMX2 25.0%, p = 0.49). The mean wound area reduction was 0.18 ± 0.48 cm^2^ per week for AMX1 and 0.15 ± 0.63 cm^2^ week for AMX2 (p = 0.42). When we compared wound healing trajectories in healers and non-healers. There were no differences in the mean wound area reduction for healers (0.26 ± 0.40 cm^2^ per week) and non-healers (0.14 ± 0.52 cm^2^ per week, p = 0.20). Our results suggested there is no difference in the incidence of healing, time to heal or incidence of infection based on weekly or biweekly application of amniotic tissue.

## Introduction

Diabetes has become a global epidemic and diabetic foot ulcers (DFUs) are one of the most common components causes that lead to infection, hospitalization, and amputation.^1–3^ Usually, DFUs that heal more rapidly are less likely to develop infections and amputations. Therefore, innovations aimed at increasing the healing rates and reducing healing time could reduce the risk of infection and amputation and improve outcomes in patients with DFUs.

Human amniotic tissue has been widely used to treat non-healing diabetic foot ulcers (DFU). There are several successful randomized controlled trials (RCTs) that evaluate weekly applications of different amniotic tissue constructs to heal diabetic foot ulcers.^4^ The objective of this pilot study was to evaluate AmnioExcel^®^ (AMX) (Integral LifeSciences, Princeton, New Jersey) to heal diabetic foot ulcers using two treatment scenarios: weekly versus biweekly application over a 12-week evaluation period. We hypothesized that there would be similar clinical outcomes in patients treated with weekly or biweekly applications.

## Methods

**Figure 1.**
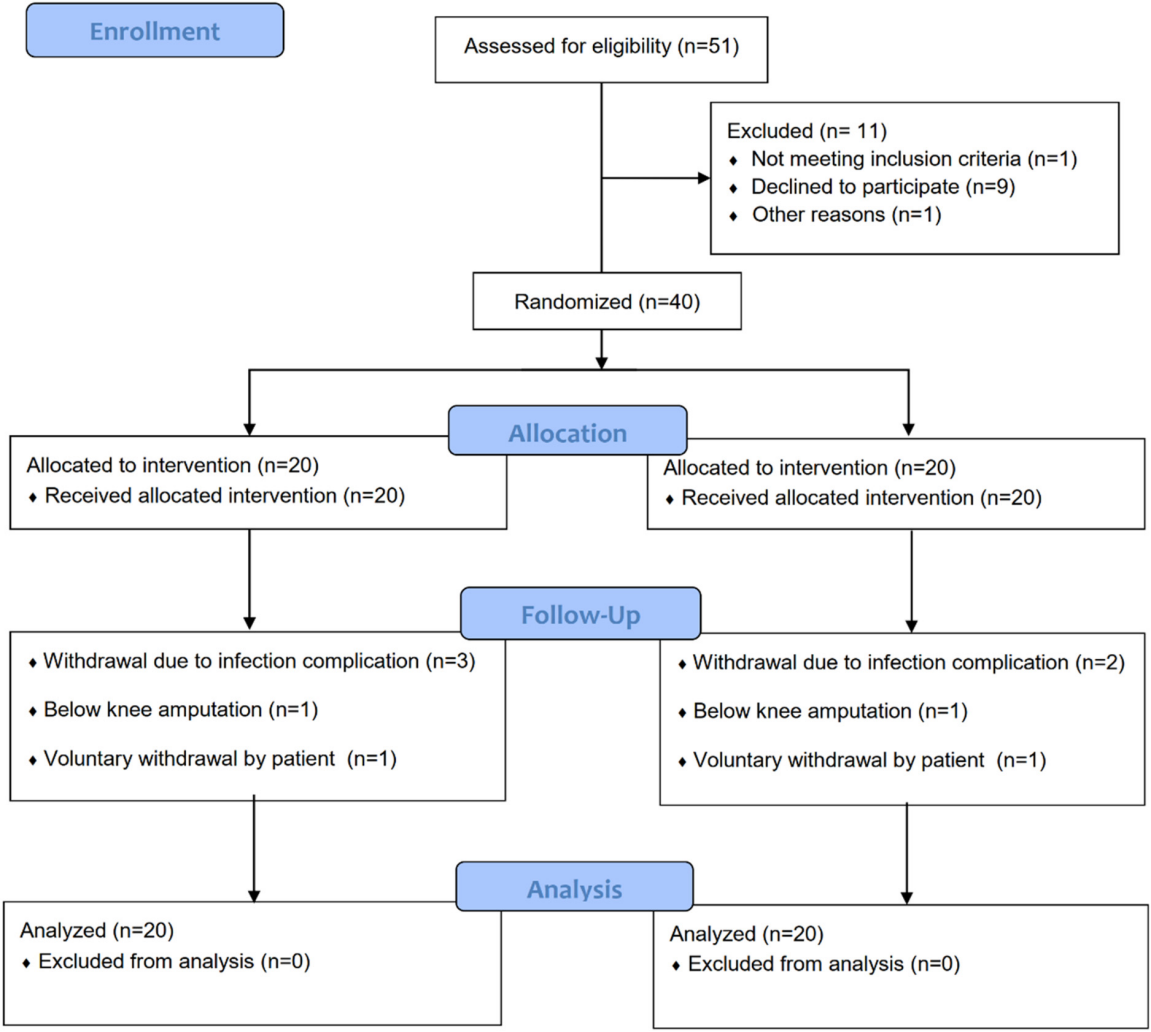
CONSORT diagram of study participants.

**Figure 2. fig2-15347346241276697:**
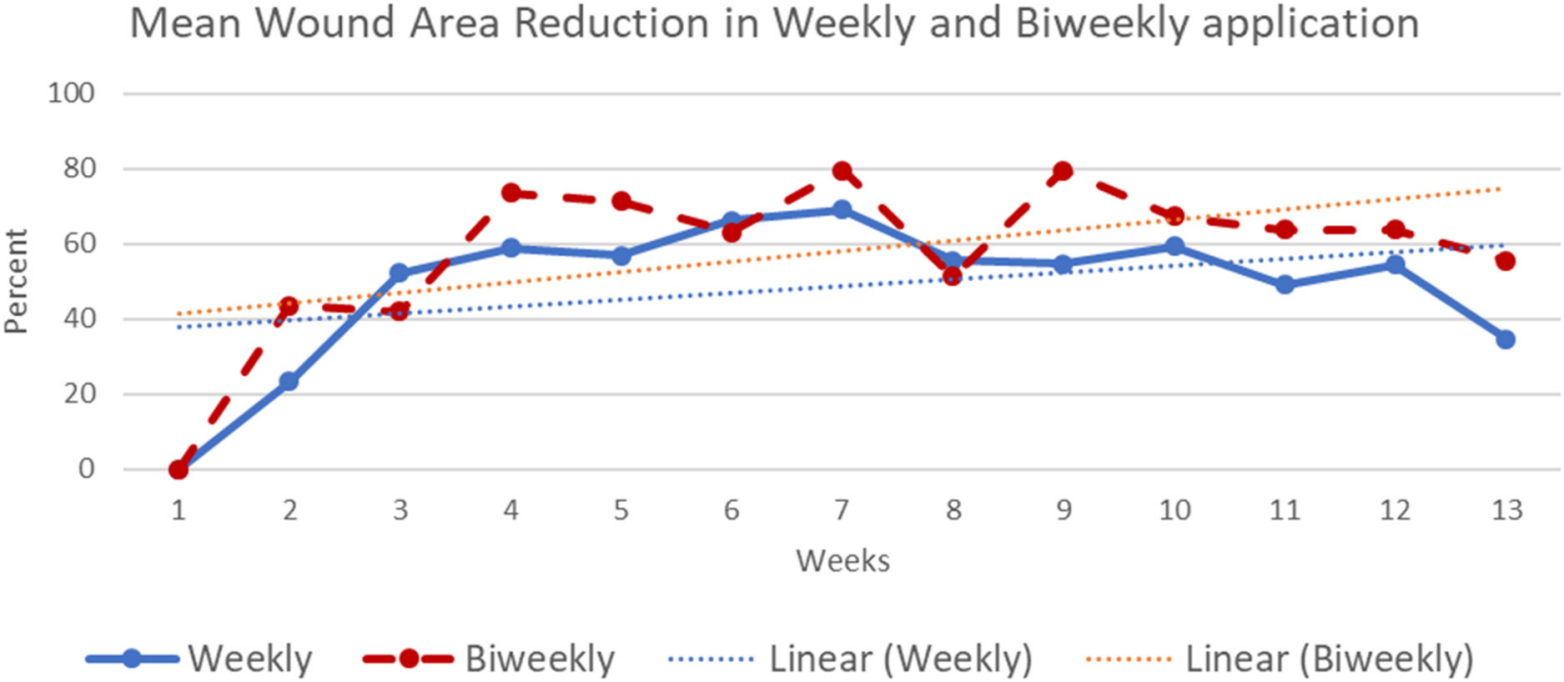
Percent mean wound area reduction over time for patients that received weekly and biweekly amniotic tissue application.

This was a randomized controlled trial approved our Institutional Review Board (STU 2019-1527) and reported on clinicaltrials.gov (NCT 04233580). We evaluated 40 patients with UT 1A and 1C^5^ ulcers for 12 weeks that received weekly (AMX1) or biweekly (AMX2) application of AmnioExcel^®^ AMX is a minimally manipulated human placental tissue consisting of dehydrated, tri-layer placental (Amnion/Chorion/Amnion) allograft. It is intended for the repair, reconstruction and replacement of skin. AMX is generally considered safe and has a low risk of side effects including pain at application site, and allergic reactions (mild), infection at infection site (rare), and pain at application site. Contraindications include active infection, known allergy to amniotic tissue and pregnancy. The study inclusion criteria were type 1 or 2 diabetes based on American Diabetes Association criteria^6^, age >21 years, and a chronic ulcer distal to the ankle present for >30 days but less than 6 months. We excluded patients with untreated osteomyelitis, gangrene (Rutherford 4–6), widespread malignancy, unhealed ischemic ulceration, or active substance abuse. We used a computer-generated randomization list to assign subjects to their group, once patients met study criteria. The CONSORT diagram is Figure 1.[Fig fig2-15347346241276697]

Sensory neuropathy was evaluated with a 10-g Semmes Weinstein monofilament and vibration perception threshold testing (VPT) (Biothesiometer, Xilas Medical Inc., San Antonio, Texas) at the great toe and medial malleolus. Sensory neuropathy was defined as either VPT >25 volts or any site missed with 10-g monofilament. Peripheral arterial disease (PAD) was defined by ankle brachial index <0.9 (ABI). We recorded systolic pressures and ankle brachial indices (ABIs) from the dorsalis pedis and posterior tibial arteries. We recorded wound area using a 3D measurement device (inSight, eKare, Fairfax, Virginia). This device has been shown to be reproducible and reliable.^7^

Ulcers were sharply debrided as deemed necessary by the treating physician and offloaded using a healing sandal (Med-Surg Post-Operative Shoe, Darco, Huntington, West Virginia) or removable cast boot (DH Offloading Walker, Össur, Reykjavík, Iceland).^8^ We defined healing as complete epithelialization with no drainage. We used the foot infection classification developed by the International Working Group on the Diabetic Foot to classify the presence and severity of infection during the follow-up.^9^

Study variables were summarized as mean and standard deviations for continuous variables and proportional comparisons for categorical variables. An intention to treat (ITT) analysis was performed. Continuous variables between groups were compared using either Student t-test or Mann-Whitney U-test, and categorical variables were analyzed using Pearson χ2 test or Fisher exact test (SPSS 27, IBM, Chicago, Illinois). This was a pilot study, so we did not have a sample size justification. We used an alpha of 0.05.

## Results

At baseline, there were no differences between the treatment groups (Table 1). The mean baseline wound area was 4.0 ± 4.9 cm^2^ for the AMX1 group and 3.3 ± 4.1 cm^2^ for the AMX2 group (p = 0.57). The mean wound area reduction (WAR) was 0.18 ± 0.48 cm^2^ per week for AMX1 and 0.15 ± 0.63 cm^2^ per week for AMX2 (p = 0.42, Figure 2). There was no difference in 50% WAR at 4 weeks based on dosing (AMX1 50%, AMX2 60%, p = 1.00). There was no difference in the survival analysis for wound healing and infection when we compared AMX1 and AMX2.

**Table 1.** Patient Demographics and Outcomes Comparison in Weekly and Biweekly Application.

	Weekly N = 20	Bi-weekly N = 20	p value
**Demographics**			
Age	58.9 ± 15.3	57.6 ± 9.8	0.81
Male	14(70)	15(75)	0.72
BMI (kg/m^2^)	32.2 ± 7.8	32.3 ± 5.0	0.98
Race/Ethnicity			
Caucasian	11(55)	9(45)	0.53
African decent	4(20)	7(35)	0.29
Hispanic	4(20)	4(20)	1.0
Other	1(5)	0(0)	1.0
Previous Ulcer	15 (75)	15 (75)	1.0
Previous Amputation	11 (55)	15 (75)	0.32
Charcot History	4 (20)	9 (45)	0.06
Diabetes Type 2	20 (100)	17 (85)	0.23
Diabetes duration (years)	15.5 ± 12.1	18.1 ± 11.1	0.79
Retinopathy	2 (10)	3 (15)	1.00
CAD	4 (20)	1 (5)	0.34
CKD I-IV	6 (30)	7 (35)	1.0
ESRD / Dialysis	4 (20)	3 (15)	1.0
CHF	3 (15)	2 (10)	1.0
Hypertension	18 (90)	16 (80)	0.66
**Sensory Neuropathy**			
Vibration Perception – foot (volts)	83.6 ± 31.0	77.3 ± 24.5	0.48
Vibration Perception – ankle (volts)	72.3 ± 31.0	68.4 ± 27.8	0.68
Abnormal monofilament	18(90)	18(90)	1.0
ABI Dorsalis Pedis	1.0 ± 0.3	1.1 ± 0.3	0.37
ABI Posterior Tibial	0.8 ± 0.4	1.0 ± 0.3	0.40
Baseline Wound Area (cm^2^)	4.0 ± 4.9	3.1 ± 4.0	0.57
**Laboratory values**			
Glycated Hemoglobin	7.4 ± 1.5	8.0 ± 2.4	0.49
White Blood Cell Count	8.8 ± 2.2	9.0 ± 3.3	0.81
CRP	24.3 ± 29.8	41.3 ± 68.4	0.62
**Off-loading**			
Removable Cast Boot	10 (50)	12 (60)	0.75
Healing Sandal	10 (50)	8 (40)	0.53
**Outcomes**			
50% Wound area reduction 4 week	12 (60)	12 (60)	1.0
Wound area reduction (cm2 week)	0.18 ± 0.48	0.15 ± 0.63	0.42
Healed (%)	5(25)	10(50)	0.19
Time to heal (days)	69.3 ± 30.3	45.8 ± 25.6	0.15
Infection	6(30)	5(25)	0.72
Time to infection (days)	50.2 ± 16.2	19.6 ± 9.1	<0.01

There were no differences in clinical characteristics, demographics or outcomes except for time to infection.

Mean and standard deviation (SD) presented for continuous variables. Categorical variables presented as N (%).

BMI = Body Mass Index, CAD = Coronary Artery Disease, CKD = Chronic Kidney Disease, ESRD = End-Stage Renal Disease, CHF = Congestive Heart Failure, ABI = Ankle Brachial Index, CRP = C-Reactive Protein.

**Figure 3.**
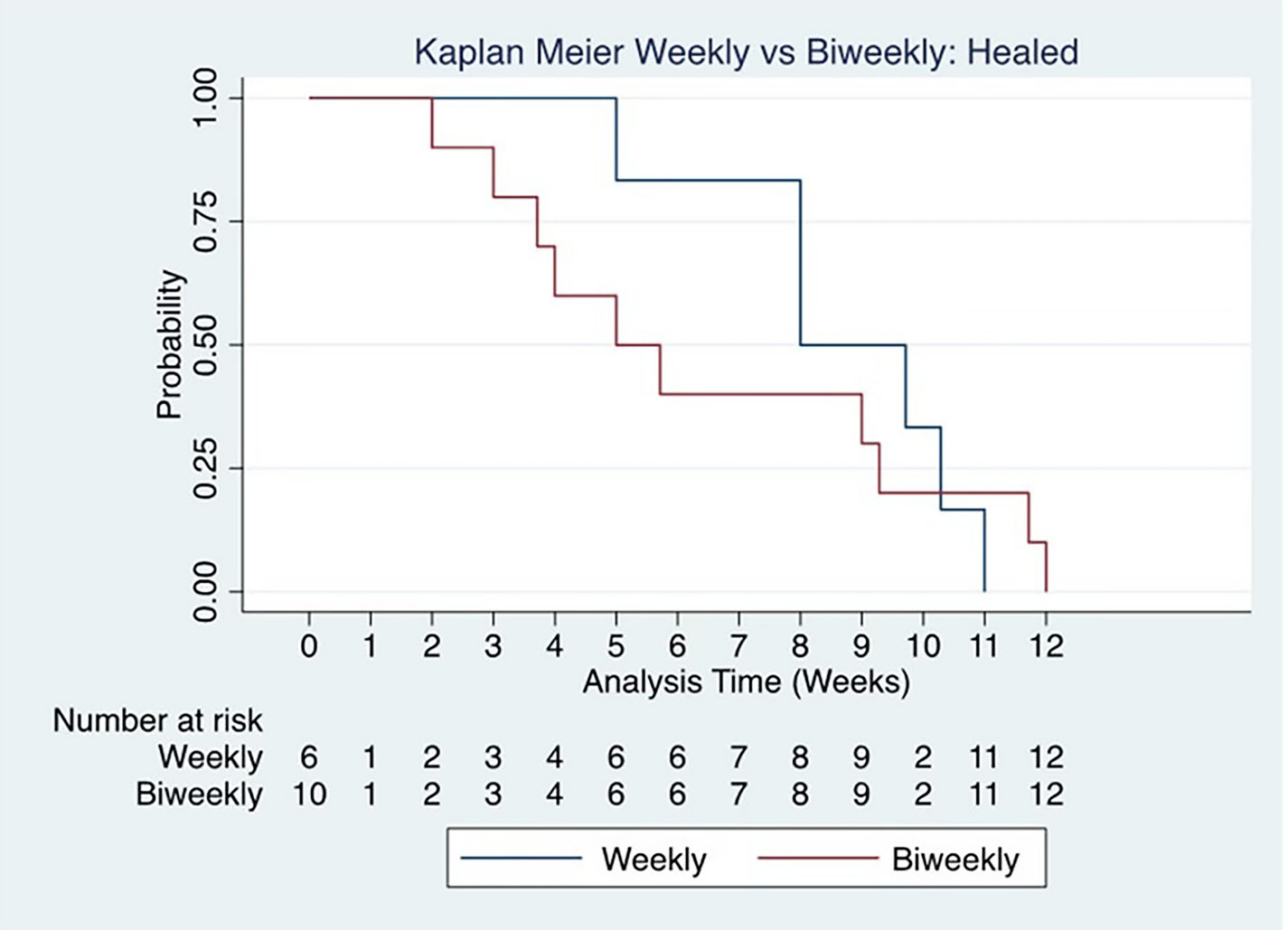
Kaplan Meier graph represents the time until ulcer healed during the study period. X-axis stands for weeks. Patients were treated either with weekly dressing (gray line) versus biweekly dressing (black line). There was no significant difference in the time to heal.

**Figure 4.**
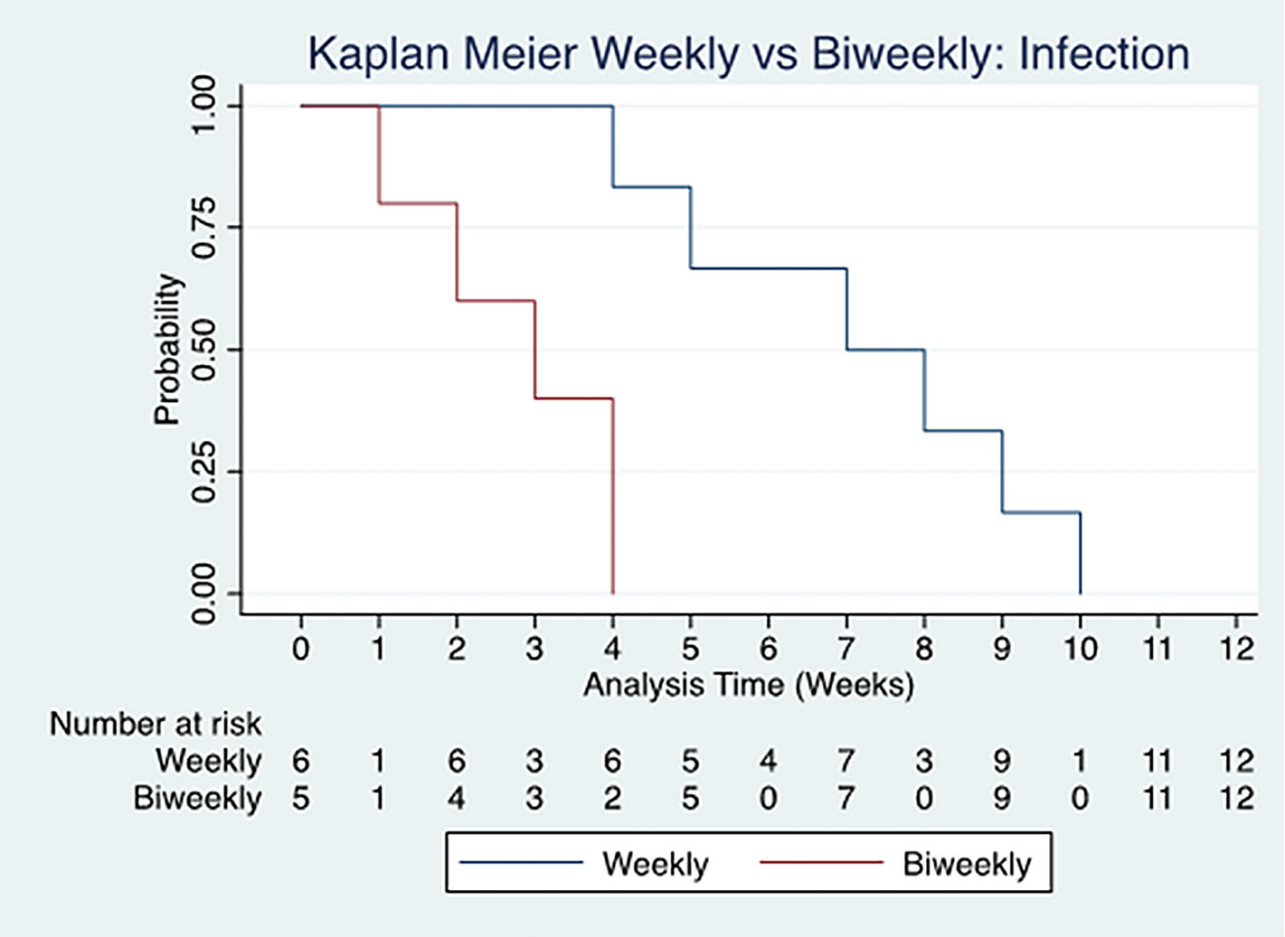
Kaplan Meier graph represents the time until infection over the course of the study period. X-axis represents days. Patients were either treated with weekly dressing (gray line) versus biweekly dressing (black line).

There were no differences in the incidence of healing (AMX1 30.0% vs AMX2 50.0%, p = 0.20), time to heal (AMX1 69.3 ± 30.3 days AMX2 45.8 ± 25.6 days, p = 0.15), and the incidence of infection (AMX1 35.0% vs AMX2 25.0%, p = 0.49). There was a significantly longer mean time to infection in people with weekly application (AMX1 50.2 ± 16.2 days, AMX2 19.6 ± 9.1 days, p ≤ 0.01). Figures 3 and 4 are Kaplan Mier survival analysis for the two treatment arms based on weekly or biweekly application of amniotic tissue for time to heal and time to infection. There was not a significant difference in time to heal (p = 0.15) or time to infection (p = 0.14).

**Figure 5.**
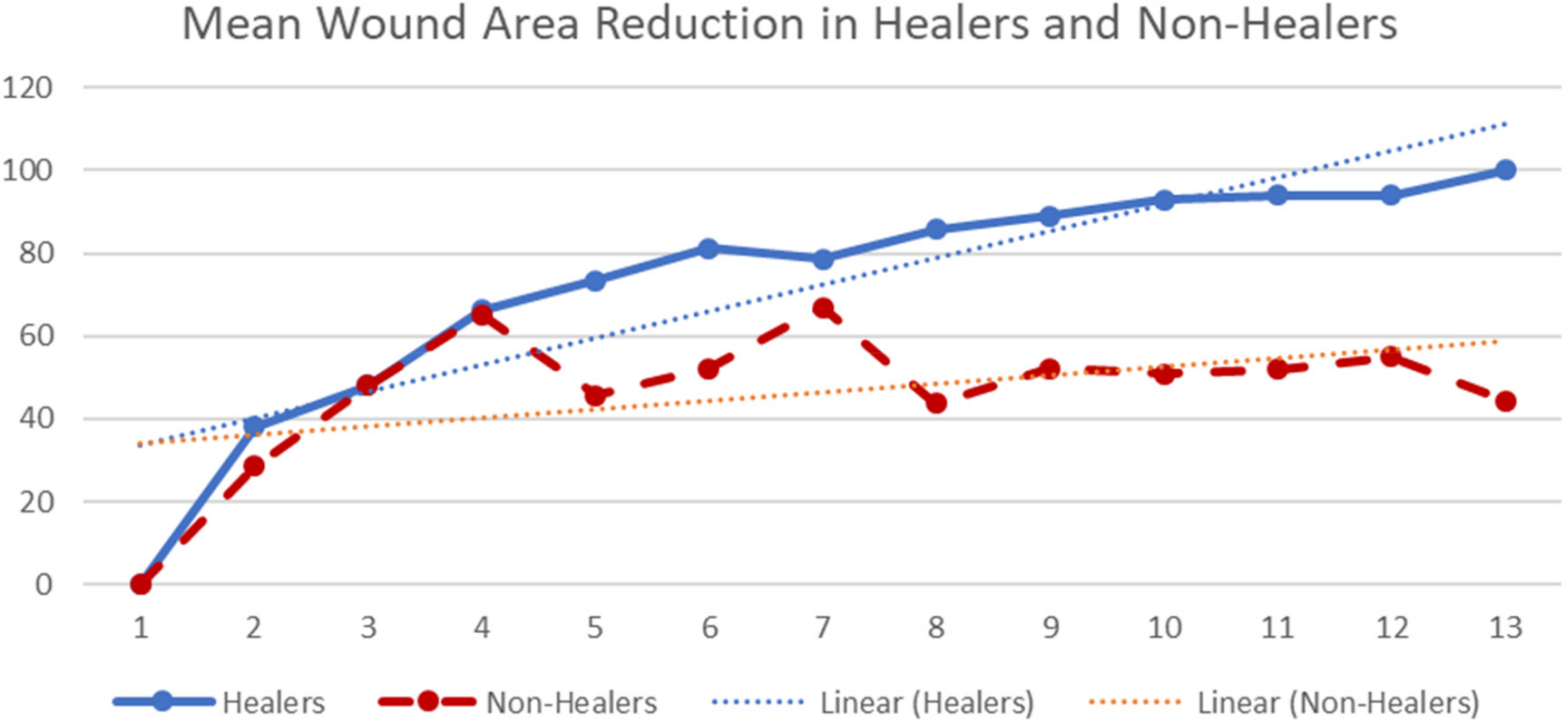
Percent wound area reduction over time for healers compared to non-healers. The wound area reduction was essentially identical among healers and non-healers until week 5.

When we compared wound healing trajectories in healers and non-healers during the 12-week evaluation period, there was no difference in baseline wound area for healers (3.4 ± 4.2 cm^2^) and non-healers (3.7 ± 4.7 cm^2^, p = 0.83, Table 2, Figure 5). There were no differences in the mean WAR for healers (0.26 ± 0.40 cm^2^ per week) and non-healers (0.14 ± 0.52 cm^2^ per week, p = 0.20). At four weeks, the healers had significantly more patients with 50% WAR compared to non-healers (healers 93.3%, non-healers 40.0, p ≤ 0.01). Infection was significantly more common in non-healers (healers 6.7%, non-healers 40.0, p ≤ 0.01). There were 11 people with infections. One subject healed (6.7%) and 10 (40.0%) did not heal (p = 0.01). Of the 10 patients that did not heal, 5 subjects withdrew from the study due to infection, and 5 subjects were treated for infection and completed the study (Figure 1).

**Table 2.** Patient Demographics and Outcomes Comparison in Healers and Non-Healers.

	Healed N = 15	Not Healed N = 25	p value
**Demographics**			
Age	57.9 ± 14.3	58.8 ± 11.9	0.83
Male	11 (73.3)	18 (72.0)	0.67
BMI (kg/m^2^)	30.7 ± 6.2	33.3 ± 6.7	0.23
Previous Ulcer	12 (80.0)	18 (72.0)	0.57
Previous Amputation	12 (80.0)	14 (56.0)	0.28
Charcot History	6 (40.0)	7(56.0)	0.58
Diabetes Type 2	15 (100.0)	22 (88.0)	0.31
Diabetes duration (years)	16.3 ± 11.9	17.3 ± 11.5	0.80
Retinopathy	2 (13.3)	3 (12.0)	0.28
CAD	2 (13.3)	3 (12.0)	0.28
CKD I-IV	7 (46.7)	6 (24.0)	0.14
ESRD / Dialysis	3 (20.0)	4 (16.0)	0.75
CHF	2 (13.3)	3 (12.0)	0.90
Hypertension	13 (86.7)	21 (84.0)	0.82
**Sensory Neuropathy**			
Vibration Perception – foot (volts)	80.5 ± 28.2	80.6 ± 28.4	0.98
Vibration Perception – ankle (volts)	71.7 ± 28.0	69.5 ± 30.3	0.82
Abnormal monofilament	15 (100.0)	21 (84.0)	0.31
ABI Dorsalis Pedis	1.1 ± 0.3	1.0 ± 0.3	0.24
ABI Posterior Tibial	1.0 ± 0.3	0.9 ± 0.4	0.60
Baseline Wound Area (cm^2^)	3.4 ± 4.2	3.7 ± 4.7	0.83
**Laboratory values**			
Glycated Hemoglobin	7.7 ± 2.9	7.7 ± 1.3	0.96
White Blood Cell Count	9.6 ± 3.5	8.4 ± 2.2	0.23
CRP	39.8 ± 63.2	28.9 ± 46.9	0.54
**Off-loading**			
Removable Cast Boot	9 (0.6)	14 (56.0)	0.80
Healing Sandal	6 (40.0)	11 (44.0)	0.80
**Outcomes**			
50% Wound area reduction 4 week	14 (93.3)	10 (40.0)	<0.01
Wound area reduction (cm^2^ week)	0.26 ± 0.40	0.14 ± 0.52	0.20
Infection	1 (6.7)	10 (40.0)	0.01
Weekly AM/XL application	6 (40.0)	10 (40.0)	0.20

There were no differences in clinical characteristics, demographics or outcomes except for the incidence of infection. People that did not heal were more likely to have an infection during the study.

Mean and standard deviation (SD) presented for continuous variables. Categorical variables presented as N (%).

BMI = Body Mass Index, CAD = Coronary Artery Disease, CKD = Chronic Kidney Disease, ESRD = End-Stage Renal Disease, CHF = Congestive Heart Failure, ABI = Ankle Brachial Index, CRP = C-Reactive Protein.

## Discussion

The results of this study indicated that there were no differences in outcomes based on using amniotic tissue every week or every other week for 12 weeks. The intent to treat results were similar to several other DFU RCTs that used amniotic tissue^4^ or other cellular and tissue-based products.^10^ Most of the RCTs in this area use weekly applications; however biweekly applications have also been found to be effective. Biweekly application of human amniotic allograft versus standard of care were compared in two randomized trials and demonstrated higher rates of WAR and complete healing^11,12^. RCTs have reported a higher incidence of healing with weekly compared to biweekly application of amnion allograft in diabetic foot ulcers. For instance, Zelen et al reported that that weekly applications healed faster (2.4 vs 4.1 weeks p = 0.039), and there was a higher proportion of DFUs that healed (90% weekly vs 50% biweekly).^11^ Furthermore, Gentzkow et al compared cultured human dermis allograft to standard of care and found a dose-response relationship; weekly application demonstrated faster healing and higher incidence of completely healed DFUs (50.0%) than in the biweekly group (21.4%).^13^ When selecting graft treatment frequency in the office setting, cost is important to consider, and interestingly in the Gentzkow study, the same total number of grafts^8^ were used in each of the weekly and biweekly groups.

There should be a distinction between products that are designed for serial application such as bioengineered tissue (bioengineered tissue from neonatal foreskin), synthetic materials (polyurethane dermal matrix), xenografts (porcine intestine or bovine tissue) or placental tissue and other products that can be used with one application. The construct of single application products is more robust and can be sutured or stapled into the wound. These are often left in place for several weeks. There are placental tissue, bovine matrix, and composite/synthetic products that have been reported in the medical literature for diabetic foot wounds that use a single application. For instance, diabetic foot ulcer RCT's with bilayer xenografts^14^ and dermal matrix bovine graft^15^ demonstrated a higher incidence of healing and faster healing compared to standard of care. Driver and colleagues reported an average number of applications of 1.0 and a healing incidence of 51% versus 32%. Lantis and colleagues found that an average of 1.4 applications over a 12-week period of a dermal matrix bovine graft demonstrated a higher incidence of healing compared to daily wet-to-dry dressings (45.6% vs 27.9%) in DFUs.^14,15^

It is common to use WAR at four weeks as a surrogate marker to define wound healing. Often 50% WAR is used based on work from Sheehan.^16,17^ or 60% WAR for complex wound based on work from Lavery and colleagues.^18^ Patients with weekly and biweekly both had the same proportion of subjects with 50% WAR. Among healers 93.3% achieved this milestone compared to 40% of people that did not heal.

## Limitations

This was a pilot study, so it was expected to be underpowered. A larger, adequately powered study is needed to support definitive conclusions. There was a small difference in the incidence of wound healing in the two treatment approaches. If a study was powered based on the incidence of healing of 30.0% and 50.0% with a power of 0.80, and an alpha of 0.05, 93 subjects would be required in each treatment arm.

## Conclusion

There were no differences in clinical outcomes between weekly and biweekly application of amniotic tissue in the intent to treat analyses. There were no differences in the incidence of healed ulcers, the time to healing, infections, 50% WAR at four weeks and wound healing trajectories when patients were randomized to receive weekly or biweekly treatment. There is a need for additional comparative study of the frequency of graft dosing to determine optimal effectiveness in the treatment of DFUs.
